# Mindfulness and compassion-based programs on eating behavior of post-bariatric surgery patients: A two phased clinical trial protocol

**DOI:** 10.1016/j.mex.2024.102885

**Published:** 2024-08-09

**Authors:** Erika Blamires S. Porto, Jesus Montero-Marin, Luiz Gustavo Quadros, Jean Kristeller, Vicente Sarubbi Junior, Luis Augusto Mattar, Javier Garcia-Campayo, Marcelo Demarzo

**Affiliations:** a Mente Aberta – Brazilian Center for Mindfulness and Health Promotion. Department of Preventive Medicine at Escola Paulista de Medicina, Universidade Federal de São Paulo, Rua Botucatu, 740, Zip code: 04023-062. São Paulo, SP, Brazil; b Department of Psychiatry, University of Oxford, Oxford, OX37JX, UK; c Kaiser Day Hospital, Rua Quinze de Novembro, 3975, Zip code: 15015-110, Sao Jose do Rio Preto, SP, Brazil; d Department of Psychology, Indiana State University, 200 N 7th St, Terre Haute, IN, 47809, USA; e Department of Medicine, Universidade Estadual de Mato Grosso do Sul, *Av*. Dom Antonio Barbosa (MS-080), 4.155, Zip Code: 79115-898, Campo Grande, MS, Brazil; f LEV Clinic, Avenida Vasconcelos Costa, Bairro, 967 — 10° andar — Osvaldo Rezende, Zip code: 38400-450, Uberlândia, MG, Brazil; g Department of Psychiatry, Miguel Servet Hospital, Aragon Institute of Health Sciences, Calle Gonzalo Calamita, 50009, Zaragoza, Zamora, Spain; h Teaching, Research & Innovation Unit, Parc Sanitari Sant Joan de Déu, Sant Boi de Llobregat, Barcelona, Spain; i Consortium for Biomedical Research in Epidemiology & Public Health (CIBER Epidemiology and Public Health - CIBERESP), 28029 Madrid, Spain

**Keywords:** Bariatric surgery, Weight gain, Feeding behavior, Mindfulness, Compassion, Randomized controlled trial

## Abstract

Introduction: Weight regain after bariatric surgery remains a relevant and worrisome topic, requiring greater understanding and involvement in research into new adjuvant treatments. This study aims to compare the preliminary effectiveness and feasibility of the Mindfulness-Based Health Promotion and Attachment-Based Compassion Therapy programs as opposed to usual treatments (workshops) on the eating behavior of patients with progressive weight gain after bariatric surgery in Brazilian patients at a private clinic. It was hypothesized that both interventions are feasible and that the self-compassion program may be more effective than the mindfulness program. Methods: The study will be divided into two phases: a cross-analytical study of those who underwent bariatric surgery and a randomized controlled trial only with the ones who had weight regain. Interventions will be conducted for eight weeks synchronously with three assessment points (baseline, post intervention, and 6-month follow-up), both online. The primary outcome will be a change in eating behavior. Secondary outcomes will include improved quality of life, enhanced body image satisfaction and reduced distortion (Brazilian Silhouette Scales for adults), better weight management (maintenance or weight reduction), increased frequency of activity and monitoring with the surgery team. Qualitative data will also be collected by online identification of a sub-sample of participants. Results: Improvements are expected in eating behavior, weight, reverse progressive weight gain, classification of self-image, quality of life, and levels of mindfulness, self-compassion, and anxiety. Conclusion: This study seeks to gather preliminary evidence on the effectiveness of mindfulness and compassion training for the adjunctive treatment of progressive weight gain in post-bariatric patients.

*Clinical Trials.gov* Registration ID: NCT04171713.

## Graphical abstract

**Figure:**
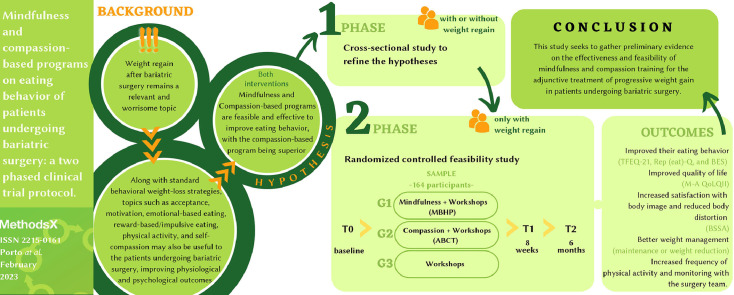

Specifications table

**Table:** 

Subject area:	Medicine and Dentistry
More specific subject area:	Mindfulness and eating behavior
Name of your protocol:	Mindfulness and compassion-based programs for patients undergoing bariatric surgery
Reagents/tools:	N.A.
Experimental design:	This is a mixed methods study (quantitative-qualitative) divided into two phases: a cross-sectional study (phase 1) to refine the hypotheses and a randomized controlled feasibility study (phase 2).
Trial registration:	Clinicaltrials.gov: NCT04171713
Ethics:	This study was approved by the Research Ethics Committee from the local Institutional Review Board, under the CAAE protocol reference number 18529519.5.0000.5505. Participants will sign the Informed Consent Form online and data confidentiality will be guaranteed.
Value of the Protocol:	• First randomized controlled feasibility study with people who underwent bariatric surgery and regained weight on mindfulness and self-compassion. • Intervention with two different approaches and three assessment points studding five facets of the eating behavior (baseline, post intervention, and 6-month follow-up). • Improvements are expected in eating behavior, reversing progressive weight gain, classification of self-image, quality of life, and in levels of mindfulness, self-compassion, and anxiety.

## Introduction

Obesity is a public health problem, especially in developing countries, where 85 % of worldwide premature deaths from noncommunicable diseases occur [1]. Brazil is following the global trend, with 20,3% obese men and 22,6% obese women, according to the latest national survey [2]. For those with Obesity class III, e.g. with the Body Mass Index (BMI) ≥ 40 Kg m^-1^², or those with the BMI between 30 and 35 kg m^-1^² (Obesity class II) in the presence of clinical comorbidities, bariatric surgery still offers better results than non-surgical treatments, which have short-term benefits in metabolic markers and comorbidity reduction [[3], [4], [5], [6], [7]]. Usually, during the first years after bariatric surgery, patients tend to be more compliant with the recommendations regarding diet and lifestyle. Then, their compliance naturally decreases with time, which is reflected by body weight gain and the resultant deterioration in quality of life [8,9] Sustainable weight loss in long-term follow-up remains, however, a complex and relevant topic in the bariatric field [5].

Weight regain greatly affects health, causing economic repercussions on the management of ongoing obesity and the return of comorbidities [4]. This weight loss-and-regain cycle can also be psychologically devastating [4], inducing feelings of disappointment, frustration, fear of returning to their heaviest weight, and shame [10]. Weight regain has mental suffering related aspects as well, including depression and anxiety [11]. Moreover, body image dissatisfaction has been related as a sensitive indicator of low psychological well-being after surgery [12]. This whole set of factors, and others, can lead to constant contact with negative emotions.

This issue has no simple solution [6]. The inability to permanently change behavioral responses to environmental conditions and pressures hinders successful treatments [7]. Several of overweight or obese individuals eat compulsively in response to negative emotions [13]. Among the bariatric population with weight regain, studies report other additional compulsion aspects related to eating, such as binge eating, night eating, grazing, and uncontrolled eating [8,11,14,15]. The increase in lack of control eating seems to increase weight regain and to decrease the perception of quality of life components [16].

Along with standard behavioral weight-loss strategies, topics such as acceptance, motivation, emotional-based eating, reward-based/impulsive eating, physical activity, and self-compassion may also be useful to the post bariatric surgery patient, improve physiological and psychological outcomes [15]. The third wave of behavioral psychotherapies could help post-bariatric patients improve since it is essential for the development of modern psychotherapy, characterized by new themes including metacognition, cognitive fusion, emotions, acceptance, mindfulness, self-compassion, dialectics, spirituality, and therapeutic relationship [17]. Among these, this study emphasizes the mindfulness-based and compassion-based programs, which have gathered increased interest over the last three decades [18], particularly from those who work on health promotion and self-compassion.

The main goal of Mindfulness-Based Programs (MBPs) is to develop autonomy, self-efficacy and empowerment of people through the capacity generated by training in mindfulness, aspects considered fundamental in the modern concept of health promotion [19]. On the other hand, compassion-based interventions (CBIs) seeks to affect empathic processes and prosocial emotions, including decreased self-criticism, decreased rumination, reception of negative life events, and regulation of emotions [19].

Although both MBPs and CBIs training programs contain meditative practices of attentional and constructive families, MBPs have a greater amount of attentional practices while CBIs have more of constructive practices, thus differing structurally in the specific cognitive mechanisms that impact on well-being.

‘Attentional’ refers to the systematic training of the intentional ability to initiate, direct and/or sustain attentional processes, strengthening the ability to be aware of the processes of thinking, feeling and perceiving, thus developing their cognitive mechanisms of attention regulation and meta-awareness. In contrast to mindfulness practices, which often focus on simply monitoring cognitive and affective patterns, ‘constructive’ meditations involve systematically altering the content of thoughts and emotions, strengthening psychological patterns that promote well-being, by developing coping mechanisms as perspective taking (process of considering how one or another would think or feel in a particular situation) and re-appraisal (process of changing how one thinks or feels about situations and events in such a way that one's response to them is altered) [20].

CBIs produced larger effects for eating behavior and rumination outcomes compared with control interventions [21]. Higher levels of self-compassion and practice of attention and acceptance strategies have improved individuals’ desire to ignore cravings for food [22], indicating that training can help monitor and inhibit eating, which then increases happiness and psychological resilience [23].

Mindfulness-Based Programs have achieved positive treatment effects. Evidence supports its use for obesity-related eating behaviors, including binge eating, emotional eating, and external eating, suggesting moderate to largely effective outcomes on weight loss [24]. On the bariatric population, there is only one pilot study that used an approach with elements of Mindfulness-Based Stress Reduction (MBSR), Mindfulness-Based Eating Awareness Training (MB-EAT), and Mindfulness Self Compassion (MSC) for ten weeks to test the feasibility, acceptability, and effectiveness of this approach compared to a standard intervention, concluding that the approach was highly achievable and acceptable to patients and also effectively decreased emotional intake, but not reverse weight gain [25]. Mindfulness training is not yet part of routine care for bariatric patients [15], neither is compassion training. Additional research is needed to determine whether these strategies are effective in the long term and whether they can be routinely introduced into the clinical practice [26]. No previous studies have directly compared specific CBIs with conventional MBIs effects on progressive weight regain.

The aims of the present study are twofold: to describe the protocol for both (1) the cross-sectional study to refine the hypotheses and (2) a Randomized Controlled Trial (RCT) to compare the preliminary effectiveness and feasibility of the Mindfulness-Based Health Promotion (MBHP) and Attachment-Based Compassion Therapy (ABCT) programs regarding the usual care on the eating behavior of patients with progressive weight gain after bariatric surgery. It was hypothesized that both interventions are feasible and effective to improve eating behavior, but the compassion-based program is superior to the mindfulness-based program because it addresses specific psychological skills (such as self-compassion) that mediate the effects on eating behavior and well-being.

## Methods

### Study design

This is a mixed methods study (quantitative-qualitative) divided into two phases: a cross-sectional study (phase 1) to refine the hypotheses and an RTC (phase 2). The report of this study will be following the STROBE Statement for Cross-Sectional Studies and the Consolidated Standards of Reporting Trials (CONSORT). The study's headquarters will be a private clinic specialized in obesity treatments located in Uberlândia, Minas Gerais State, Brazil, founded in 2011. About 8000 bariactric surgerys have been conducted on the clinic.

### Phase 1: cross-sectional study

#### Study design

This is a cross-sectional study to be conducted with patients who underwent bariatric surgery until 2020, seeking to identify the main differences between patients with weight maintenance, with expected weight gain and progressive weight regain, and thus refine the hypotheses of the study's Phase 2.

#### Participants

Participants will be included in the sample: adults aged ≥18 who underwent sleeve gastrectomy or Roux-en-Y Gastric Bypass, after at least two years. The sample considers patients with ‘weight maintenance’, those who keep the minimum weight achieved after bariatric surgery (nadir); patients with ‘expected weight gain’, a recovery <20 % of weight lost in the long term, according to the Brazilian Society of Bariatric and Metabolic Surgery (SBCBM) [27] and patients with ‘progressive weight regain’ those who recovery > 20 % of in the long term.

#### Measurements

All data will be collected by patient report using an electronic online form (SurveyMonkey®) containing closed and open questions distributed among a socio-demographic survey and validated scales in Portuguese.

- Sociodemographic and others: An instrument containing, age, gender, marital status, associated diseases, lifestyle habits, and weight evolution before and after surgery and other questions related to objectives, such as questions about adherence to post-surgical follow-up consultations, nutritional recommendations and attendance to support groups, psychological and/or psychiatric treatment and physical activity.
- The 21-item Three-Factor Eating Questionnaire (TFEQ-R21): This scale has 21 items that assess three aspects of eating behavior: cognitive restriction, emotional eating, and uncontrolled eating. Higher scores indicate greater cognitive restraint, uncontrolled eating or emotional eating [28,29].
- Repetitive Eating Questionnaire [Rep(eat)-Q]: This is a 12-item questionnaire to assess grazing, with two graze subtypes: repetitive eating and compulsive grazing. An overall average of the 12 items will be calculated. The scores for each domain will be calculated as an average of all items in each domain. Higher scores indicate more presence of grazing feeding behavior [30].
- Binge Eating Scale (BES): This is a scale of 16 items and 62 statements that was built in three stages: characteristics of binge eating, severity of each characteristic, and an external criterion of severity (frequency, amount of food, and degree of emotion involved in an episode). Each statement corresponds to a number of points from 0 to 3, ranging from absence “0″ to maximum severity “3″ of binge eating. The overall score will be categorized as follows: non-binging ≤ 17 points, moderate binging 18 to 26 points, and severe binging ≥ 27 points. [31,32].
- -Moorehead-Ardelt Quality of Life Questionnaire II (M-A QoLQII): This is a self-administered questionnaire which addresses quality of life, updated from a part of the Bariatric Analysis and Reporting Outcome System. It contains six questions with a 10-point Likert scale that assesses the following domains: general self-esteem, physical activity, social contacts, job satisfaction, pleasure related to sexuality, and focus on eating behavior. The scores for each item and the overall average of the 6 items will be used to generate an overall quality of life score. Higher scores indicate better self-perception of quality of life. [33].
- Brazilian Silhouette Scales for Adults (BSSA): This scale has 15 images for each gender, with a white figure centered on a black background. The adult scales include the BMI averages corresponding to each figure ranging from 12.5 to 47.5 kg m^-12^, with a constant difference of 2.5 points [34]. This scale will be used to assess body image distortion, when the participant indicates which image, they resemble the most (considered distorted body image when they choose a silhouette different from their current BMI) and to assess body image dissatisfaction when the participant responds what image would they like their bodies to be. Dissatisfaction with body image will be analyzed based on the disagreement between the current silhouette and what they would like to look like, and the participants will be classified as satisfied and dissatisfied. Then, subjects with body dissatisfaction will be dichotomized into those who wanted to increase their body size (dissatisfaction due to thinness) and those who wanted to reduce their body size (dissatisfaction due to excess weight).
- Mindful Attention Awareness Scale (MAAS): The MAAS is an instrument that assesses mindfulness and consciousness levels according to time. This 15-item scale indicates how often the participants experienced each of the situations described in the items. Higher scores reflect higher levels of dispositional attention [35,36].
- Self-Compassion Scale – Short Form (SCS-SF): The brief 12-item SCS-SF assesses the feeling of compassion for the self in dimensions of self-kindness, self-judgment, common humanity, isolation, mindfulness, and over-identification. Higher scores mean more self-compassion. [37,38].
- Hospital Anxiety and Depression Scale (HADS): This 14-item scale measure signs of anxiety and depression. It has three stages for the two subscales: possible anxiety and depression disorders (8–10 points), probable anxiety and depression disorder (11–14 points), and severe disorder (15–21 points) [39,40].
- Experiences in Close Relationship Scale – Reduced (ECR-R-Brazil): This 10-item scale measures adult attachment in the context of love relationships, containing two dimensions of attachment, anxiety and avoidance. Higher scores indicate higher levels of attachment-related anxiety and avoidance [41,42].

#### Study size

The sample size of 38 participants per year of surgery (2013–2019) was calculated based on the expected 0.1 % prevalence of bariatric surgery with a 95 % confidence interval and a 1 %, margin of error totaling 266 participants.

#### Statistical methods

All variables will be described using the Kolmogorov-Smirnov test to verify the normal distribution of data. For sociodemographic and other questions, absolute and relative frequencies will be calculated for categorical variables and means whereas standard deviations will be estimated for continuous variables. Pearson's and Spearman's correlation coefficients will be used to identify the correlation between the study variables. The theoretical behavior expected from the scales will be confirmed by linear regression analysis. The level of significance will be 5 % (*p* < 0.05) in all tests and no adjustments for multiple comparisons will be considered, in accordance with the exploratory nature of Phase 1.

### Phase 2: clinical trial

#### Trial design

This is a randomized controlled clinical trial of mixed methods with random allocation in three groups: G1 (MBHP + workshops); G2 (ABCT + workshops); and G3 (workshops), with patients who underwent gastroplasty and regained weight from the clinic already mentioned.

#### Eligibility criteria

Samples will consist of adults who underwent any type of bariatric surgery and had any progressive weight gain. Since the literature has no conceptual definition of weight regain [43], and taking into account the subjective nature of the issues involved in weight regain, knowing that, for some people, regaining weight (as little as possible) can be a devastating experience that contributes to a negative spiral in weight control [10], the adopted criterion will consider any weight increase from nadir. Those who by chance use medications for weight loss, who are pregnant or intend to get pregnant in the next eight months, who have substance abuse disorder (alcohol or drugs), severe depression, schizophrenia or psychotic disorders, who use drugs that cause cognitive loss of attention and concentration, and who have previously practiced mindfulness, meditation, yoga, or similar practices in the last six months will be excluded from the study. Patients diagnosed with severe depression by HADS scale who are not undergoing treatment will be referred to the nearest mental health service facility for evaluation and follow-up.

#### Interventions

**Fig. 1:**
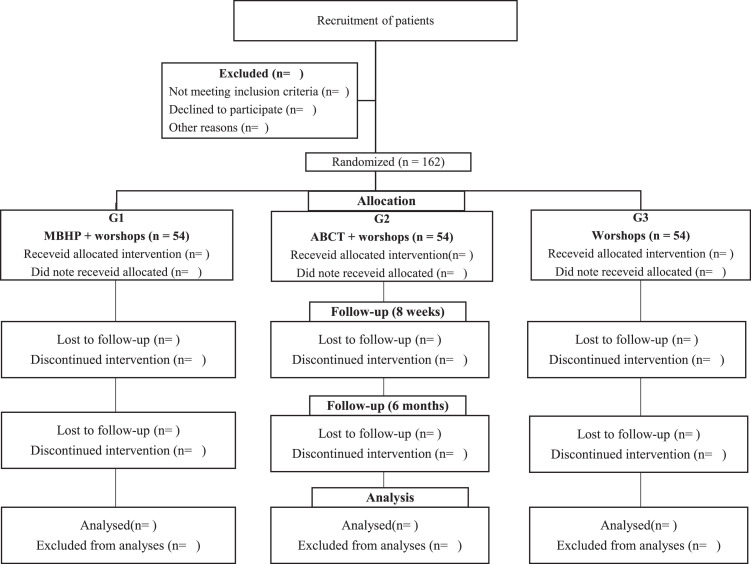
Diagram of planned study flow. Abbreviations: MBHP, Mindfulness-Based Health Promotion; ABCT, Attachment-Based Compassion Therapy.

The interventions described below will occur in an interactive online format (synchronous). Fig. 1 shows the flow diagram. Instructors of MBHP and ABCT protocols will be those health professionals (psychologist, physician or pharmacist), experienced in management of patients with severe obesity, certified by mindfulness institutions internationally validated, and with over two years of experience in facilitating groups of said protocols.

##### Workshops

All participants will be invited to two online workshops (synchronous) of two hours each, with a 30-day interval between each one. The themes and methodology of the workshops were adapted from the Instruction of the Brazilian Ministry of Health [44], which proposes several actions of food and nutrition for group work. The first meeting will work on the themes “who are we?”, “what is health for you?” and “the balance of choices”, in order to create a reflective environment and work on intrinsic motivation; the second meeting will focus on the theme “planning my food”. All participants will be invited to bring a pen and paper to write down their own reflections and plans during the workshops.

##### Mindfulness-based health promotion (+ workshops)

MBHP is a program designed by Mente Aberta Center to address universal human vulnerabilities, not focusing on any specific health condition, within the context of health promotion [45]. The program is structured and developed over eight weekly sessions, in which participants meet for an average of two hours to experience technical and conceptual learning about mindfulness (Table 1). They are also given suggestions for activities to do at home, daily. The main techniques used are practices of mindful breathing, body scan, mindful walking, and mindful movements [45].

**Table 1:** Protocols for Mindfulness-Based Health Promotion (MBHP) and Attachment-Based Compassion Therapy (ABCT) interventions.

Session	MBHP	ABCT
1	**What is mindfulness**	**Preparation for compassion**
	*Formal practice:*	*Formal practice:*
	- Raisin meditation	- Compassionate breathing
	- Body scan meditation	- Compassionate body scanning
2	**Mindfulness in breathing**	**Discovering the compassionate world itself**
	*Formal practice:*	*Formal practice:*
	- Mindful breathing	- The figure of affection: connecting with basic affection
	- Body scan meditation	- Development of a safe place
		- Compassionate action
		- Identification of the secure attachment figure
3	**Mindfulness in the body (part I)**	**Development of the compassionate world itself**
	*Formal practice:*	*Formal practice:*
	- Mindful walking	- Development of the secure attachment figure
	- Mindful breathing	- Development of a compassionate voice
4	**Mindfulness in the body (part II)**	**Understanding our relationships with compassion**
	*Formal practice:*	*Formal practice:*
	- Mindful movements	- Awareness of attachment styles
	- Mindfulness in breathing, sensations, sounds, and thoughts	- Ability to receive affection: friend, indifferent person, enemy
	- 3 min breathing in pairs	
	- Mindful walking	
5	**Mindfulness and acceptance**	**Working on ourselves**
	*Formal practice:*	*Formal practice:*
	- Mindful movements	- Show affection to friends and indifferent people
	- Mindful thinking	- Show affection to yourself
	- 3 min breathing in pairs	- Reconcile with parents
		- Three positive and negative aspects of parents
6	**Silence**	**Advanced compassion (I); forgiveness**
	*Formal practice:*	*Formal practice:*
	- Mindful movements	- Forgive yourself
	- Body scan meditation	- Ask others for forgiveness
	- Mindful breathing	- Forgive others and show compassion to enemies
	- Mindful walking	- Show forgiveness for the pain caused by a loved one
		- Recapitulation
7	**Compassion**	**Advanced compassion (II): becoming your own attachment figure and dealing with difficult relationships**
	*Formal practice:*	*Formal practice:*
	- Loving kindness (for yourself and others)	-Working with envy
	- Mindful breathing	-Becoming your own attachment figure
	- Mindful movements	- Dealing with difficult relationships
8	**Mindfulness for life**	**Beyond compassion: equanimity**
	*Formal practice:*	*Formal practice:*
	- Loving kindness (for yourself and others)	- Equanimity I: we are all equal
	- Mindful poetry	- Equanimity II: the illusion of categories
		- Equanimity III: showing the world the gratitude we were unable to return

##### Attachment-based compassion therapy (+ workshops)

ABCT is a program based on attachment styles, a psychoanalytical concept which describes the relationship children develop with their parents, and which will influence the interpersonal relationships and self-image they will eventually develop. [19]. Participants meet for two or two and half hours at each meeting for eight weeks. ABCT teaches formal (seated meditation) and informal (during daily life) self-compassion (Table 1). Experimental exercises and discussion periods conducted in each session, together with homework, help participants learn how to be kind to themselves [19].

#### Outcomes

The primary outcome will be a change in the eating behavior (TFEQ-21, Rep (eat)-Q, and BES) at all steps. Secondary outcomes will be improved quality of life (M-A QoLQII), increased satisfaction with body image and reduced distortion (BSSA), better weight management (maintenance or weight reduction), increased frequency of physical activity, and monitoring with the surgery team. The explicative variables will be the level of mindfulness (MAAS), self-compassion (SCS-SF), anxiety (HADS), and attachment style (ECR-R).

#### Assessment

**Fig. 2:**
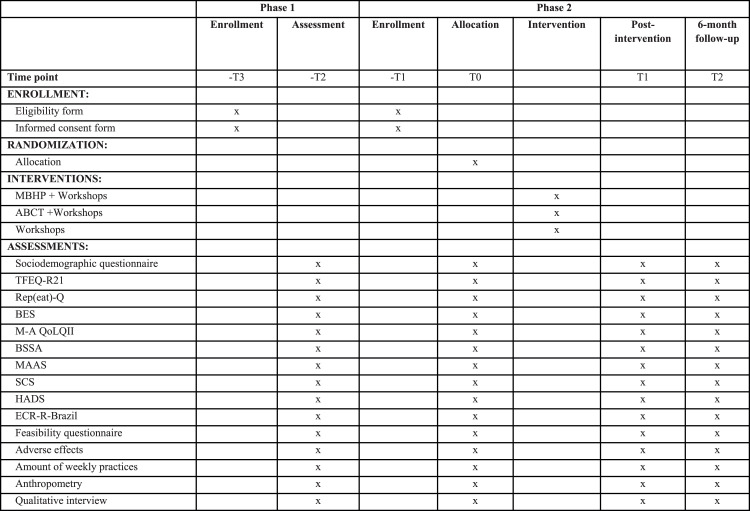
Standard Protocol Items: Recommendations for Interventional Trials (SPIRIT) diagram. Abbreviations: MBHP, Mindfulness based Health Promotion; ABCT, Attachment-Based Compassion Therapy; TFEQ-R21, The 21-item Three-Factor Eating Questionnaire; Rep(eat)-Q, Repetitive Eating Questionnaire; BES, Binge Eating Scale; M-A QoLQII, Moorehead-Ardelt Quality of Life Questionnaire II; BSSA, Brazilian Silhouette Scales for Adults; MAAS, Mindful Attention Awareness Scale; SCS, Self Compassion Scale; HADS, Hospital Anxiety and Depression Scale; ECR-R-Brazil, Experiences in Close Relationship Scale – Reduced.

Quantitative and qualitative assessments will be performed before the intervention (T0), immediately after the intervention (T1) and at the six months follow-up (T2). Participants will respond to the same electronic form used in Phase 1 of the study at the three evaluation moments (T0, T1 and T2) and after the intervention they will be able to respond subjectively about their adherence to the sessions, frequency of performing formal practices and report if there were unexpected effects (adverse events) in periods T1 and T2 by completing another electronic form [46]. After six months of follow-up, the control group will be offered the intervention (MBHP or ABCT) considered most effective to improve eating behavior (Fig. 2).

Narrative interviews will be used to produce qualitative data. Unstructured interviews will be conducted in the baseline (T0) with an open trigger question, “Tell me the story of your weight”, on which the participant will be encouraged to speak freely. In (T1) and (T2), the question “What changed in taking care of yourself?” will be added. If needed, the interviewer will direct the dialogue towards “And regarding activities of daily living?”, “And regarding food?”, and lastly “What has changed in your relationship with your body?”. The narrative interviews will be conducted online for about 30 min and statements will be recorded for later transcription and full analysis.

#### Sample size

Sample size estimates indicate that for a 30 % improvement in the primary outcome for groups G1 and G2 and 5 % for group G3 (workshop), with a 95 % bilateral confidence interval and 80 % power, each group will need at least 43 participants. Assuming that 25 % of patients would be lost to follow-up, each group would need 54 patients, with 162 participants in total.

#### Recruitment

The participants who answer the questionnaire of Phase 1 and match the criteria specific to Phase 2 will be recruited, as well as other patients that will continue to be recruited at the clinical headquarters by doctors and other health professionals from the multidisciplinary team and on the social networks of the referred clinic. It is expected that it will take 3 recruitment rounds of each intervention group to reach 54 participants per group. For better engagement during the intervention, online interaction groups will be formed for all groups (G1, G2 and G3) and participants who miss a session or a workshop will be contacted individually.

#### Data management

The information from this research will be stored in secure folders with limited access. Information from this survey will be stored in secure folders with limited access. The files will be password protected at Research Electronic Data Capture, a platform for collecting, managing and disseminating research data (REDCap), signed by the Federal University of São Paulo (UNIFESP). The list with participant numbers and personal information will be stored in a password-protected file. To avoid some risk of negligence, there will be an assignment of a data monitoring committee. Only the main investigators, or people designated by them, will have access to all the final data. Data will be stored for five years after the inclusion ends. Study participants, funders, and professionals involved will receive a summary of the study's results.

#### Analyses plan

Descriptive statistics will be used to determine the feasibility, acceptability, and effect of Mindfulness-based and compassion-based interventions. The initial baseline characteristics will be revised visually for an approximate balance between groups. For the sociodemographic questionnaire (including the different surgical procedures performed), absolute and relative frequencies will be calculated for categorical variables, whereas means and standard deviations will be estimated for continuous variables.

Statistical procedures will be performed on an intention-to-treat and/or per-protocol basis. For data tabulation and analysis, Excel 2010 programs and SPSS software version 23.0 for Windows (SPSS Inc.; Chicago, IL, USA) will be used. All variables will be subjected to the Kolmogorov-Smirnov test to verify the normal distribution of data. For initial comparisons between the groups, the unpaired *t*-test (two-tailed) or Mann-Whitney U test (two-tailed) will be used for continuous variables whereas Fisher's exact test (two-tailed) and the chi-square test will be used for categorical variables.

After the intervention, a within- and between-group comparative analysis of the scales will be performed with initial and final data using the paired and unpaired *t*-test (two-tailed) for variables with normal distribution (mean, standard deviation, and 95 *%* confidence interval) and the Mann-Whitney U test (two-tailed) for variables with non-normal distribution. Differences between the initial and final means (Δ) of the study variables will be calculated and a comparative analysis of these means will be performed using the *t*-test for independent samples or the Mann-Whitney U test. Statistical procedures will be performed per protocol based on the intention-to-treat analysis. The number needed to treat (NNT) and the number needed to harm (NNH) will be calculated.

To identify the correlation between the study variables and HADS scales, the MAAS, SCS, MAQoLQII, Rep(eat)-Q, TFEQ-R21, BES, ECR-R-Brazil, silhouette scales, and Pearson's and Spearman's correlation coefficient will be used at three time points. At the end of the study, linear regression analyses will be performed with the total sample using complete cases and stratified by weight gain to assess the mediating effect of the statistical relationship between the study scales in the intervention group.

The laminates are established from less postoperative weight in the first year, as follows: 1 to 10 % weight regained from 11 to 20 %, etc. The level of significance will be 5 % (*p* < 0.05) in all tests.

The qualitative data will be analyzed using the thematic content analysis technique [47]. The NVivo software (version 12) will be used for this organization and treatment. Data will be treated by lexical coding, thematic analysis, and association between linguistic, paralinguistic, and sociodemographic variables.

#### Research ethics approval

This study was approved by the Research Ethics Committee from the local Institutional Review Board, under the CAAE protocol reference number 18529519.5.0000.5505, in August 2019. Participants will sign the Informed Consent Form online and data confidentiality will be guaranteed.

## Discussion

To the best of our knowledge, this RCT is the first to directly compare the effectiveness of a protocol of CBI (ABCT) with an MBI (MBHP) in bariatric patients with progressive weight regain in feeding behaviors settings. Both programs in this study were developed to meet the cultural characteristics of Latin Americans [19,45] with maximum feasibility, safety and efficacy.

The present study is made relevant given the possible consequences for both physical and emotional health in the progressive weight regain [4,10], especially since training in the MBHP and ABCT protocols may increase levels of mindfulness, self-compassion and reduce levels of anxiety, as observed in other studies [18,21], and also promote improvements in quality of life, satisfaction with body image and reduced distortion. Thus, it is believed that the reduction of negative emotions or better management skills of these emotions can improve eating behaviors, mainly with the reduction in the prevalence of grazing, binge eating, cognitive restriction, emotional eating and uncontrolled eating, all of which were previously associated with weight regain and increased psychological distress [8,11,[14], [15], [16]].

Well-being improvement can mobilize better self-care engagement, for instance increasing the frequency for both monitoring with the surgery team and physical activity. Together, all the previously mentioned aspects can contribute, in medium to long term, to a better weight management (maintenance or weight reduction) throughout life.

And finally, it was hypothesized that ABCT training could be more effective than the mindfulness program because it has lots of ‘constructive’ practices and this family of practices may aim to promote healthy interpersonal dynamics, to strengthen a commitment to ethical values, or to nurture habits of perception that lead to enhanced well-being [20]. Also, because attachment orientation may be a factor worth considering when addressing disinhibited eating, especially in the context of poor outcomes following bariatric surgery [48]. If this hypothesis is confirmed, further studies will be needed to confirm whether this can be considered another strategy with potential for adjuvant treatment of post-bariatric obesity recurrence.

This study will, however, have data collection limitations since it will be conducted with a self-administered online questionnaire, which does not allow participants to discuss any questions that arise during its completion or to compare results with face-to-face interventions, besides any memory bias when reporting high and retrospective weight.

## Conclusion

This study seeks to gather preliminary evidence on the effectiveness of mindfulness and compassion training for the adjunctive treatment of progressive weight gain in post-bariatric patients.

## Authors contribution

Porto E.B.S: conception and design of the study, drafting the original article and final approval of the version to be submitted. Garcia-Campayo J. and Junior, V.S: conception and design of the study, revising it critically for important intellectual content and final approval of the version to be submitted. Kristeller J., Montero-Marin J., Mattar, L.A. and Quadros, L.G., revising it critically for important intellectual content and final approval of the version to be submitted. Demarzo, M: supervision, conception and design of the study, revising it critically for important intellectual content, and final approval of the version to be submitted.

## Financial support

This work was supported by 10.13039/501100003593National Council for Scientific and Technological Development in Brazil (grant numbers 314935/2021-5 and 88887.619511/2021-00).

## Declaration of competing interest

The authors declares that they have no known competing financial interests or personal relationships that could have appeared to influence the work reported in this paper.

## Data Availability

No data was used for the research described in the article.
